# Vertebral Bone Marrow and Endplate Assessment on MR Imaging for the Differentiation of Modic Type 1 Endplate Changes and Infectious Spondylodiscitis

**DOI:** 10.3390/jcm9030826

**Published:** 2020-03-18

**Authors:** Ursula Schwarz-Nemec, Klaus M. Friedrich, Christoph Stihsen, Felix K. Schwarz, Siegfried Trattnig, Michael Weber, Josef G. Grohs, Stefan F. Nemec

**Affiliations:** 1 Division of Neuroradiology and Musculoskeletal Radiology, Department of Biomedical Imaging and Image-guided Therapy, Medical University of Vienna, A-1090 Vienna, Austria; ursula.schwarz-nemec@meduniwien.ac.at (U.S.-N.); felixkonstantin.schwarz@gmail.com (F.K.S.); michael.weber@meduniwien.ac.at (M.W.); stefan.nemec@meduniwien.ac.at (S.F.N.); 2 Department of Orthopaedics and Trauma Surgery, Medical University of Vienna, A-1090 Vienna, Austria; christoph.stihsen@meduniwien.ac.at (C.S.); josef.grohs@meduniwien.ac.at (J.G.G.); 3 MR Center of Excellence, Department of Biomedical Imaging and Image-guided Therapy, Medical University of Vienna, A-1090 Vienna, Austria; siegfried.trattnig@meduniwien.ac.at

**Keywords:** magnetic resonance imaging, Modic type 1 changes, infectious spondylodiscitis, vertebral bone marrow edema, vertebral endplate

## Abstract

On magnetic resonance (MR) imaging, Modic type 1 (MT1) endplate changes and infectious spondylodiscitis share similar findings. Therefore, this study investigated vertebral bone marrow and endplate changes to enable their differentiation. The lumbar spine MR examinations of 91 adult patients were retrospectively included: 39 with MT1; 19 with early spondylodiscitis without abscess; and 33 with advanced spondylodiscitis with abscess. The assessment included percentage of bone marrow edema on sagittal short tau inversion recovery images, and the signal ratio of edema to unaffected bone and endplate contour (normal; irregular, yet intact; blurred; destructive) on sagittal unenhanced T1-weighted images. Differences were tested for statistical significance by Chi-square test and mixed model analysis of variance. The MR diagnostic accuracy in differentiating MT1 and spondylodiscitis was assessed by cross-tabulation and receiver-operating characteristic analysis. The endplate contours, edema extents, and T1-signal ratios of MT1 (extent, 31.96%; ratio, 0.83) were significantly different (*p* < 0.001) from early spondylodiscitis (56.42%; 0.60), and advanced spondylodiscitis (91.84%; 0.61). The highest diagnostic accuracy (sensitivity, 94.87%; specificity, 94.23%; accuracy, 94.51%) in identifying MT1 was provided by an irregular, yet intact endplate contour. This may be a useful MR feature for the differentiation between MT1 and spondylodiscitis, particularly in its early stage.

## 1. Introduction

Modic type 1 (MT1) vertebral endplate changes have been controversial findings since first defined in the late 1980s to describe endplate degeneration and subchondral bone marrow edema on MR imaging [1,2,3]. In the context of intervertebral osteochondrosis, these were initially believed to originate from mechanical disruption related to disc degeneration, resulting in increased endplate stress [1]. Lumbar disc herniation is a risk factor for developing MT1 changes, and, at the same time, MT1 can coexist with accelerated disc degeneration [4,5]. However, current data suggest an inflammatory pathogenesis, reflecting active degeneration, with an efflux of inflammatory mediators from the disc into the bone [6,7,8]. Bone marrow edema may correspond to endplate microfractures, accompanied by an increased vascularity [6,7,8]. Controversially, this micro-environment may promote superimposed low-grade infections with *Propionibacterium acnes* and *Corynebacterium propinquum* [9,10,11,12,13,14,15]. MT1 might thus represent combined overloading, inflammation, and infection, which has drawn attention to a debate about its treatment, including the use of antibiotics, bisphosphonates, and therapeutic antibodies [16,17,18,19,20].

This is where the current MR imaging study becomes relevant. Vertebral bone marrow edema is a non-specific finding associated with low back pain, including a myriad of differential diagnoses: degeneration; Schmorl nodes; trauma; inflammation or infection; or sickle cell disease, as well as an iatrogenic, neurological, or neoplastic origin [21,22,23]. Advanced infectious spondylodiscitis, with endplate destruction and height decrease, and perivertrebral abscess formation, is usually a straightforward diagnosis [24,25,26,27]. In contrast, because of the presence of bone marrow edema, the resemblance of MT1 to early spondylodiscitis, without bone destruction and abscess formation, is a diagnostic dilemma [28,29,30,31]. This issue becomes more important considering the overlap of MR findings, insidious symptoms, and etiological mechanisms between MT1 and spondylodiscitis. Shared MR features, such as abnormal discal signal intensities, mild paravertebral edema, and contrast enhancement of the bone and disc may be of limited help for differentiation, as well as endplate erosions, which are seen in osteochondrosis and infection [22,23,27,28,29,30,32]. Elevated inflammatory markers point toward spondylodiscitis, but it may be misdiagnosed as MT1 in an afebrile patient [33]. All these diagnostic ramifications underscore the necessity for strategies that could enable differentiation between MT1 and spondylodiscitis, and thus, optimize planning of CT-guided biopsy, and prevent treatment delay and failure in the patients’ outcome [34].

Consequently, this study investigated the MR imaging features of the vertebral bone marrow and endplate in patients with MT1 and spondylodiscitis, particularly in its early stage, to obtain differentiating features that would aid in diagnostic accuracy.

## 2. Materials and Methods 

This retrospective study was approved by our institutional review board (ethical code number, 1325/2017; date of approval, 25 April 2017; date of extension of validity of approval, 14 April 2019), and the study procedure was performed in accordance with the Declaration of Helsinki. Written, informed consent for clinical lumbar MR imaging was obtained from all patients, and informed consent for study participation was waived. Due to organizational reasons, the retrospective study evaluation was not started until June 2019. 

### 2.1. Study Population

For this study, we reviewed the medical records from lumbar spine MR examinations, performed between January 2016 and April 2018, of adult patients (18 years of age or older), who had been referred to our tertiary referral center to undergo work-up because of low back pain.

**Figure 1:**
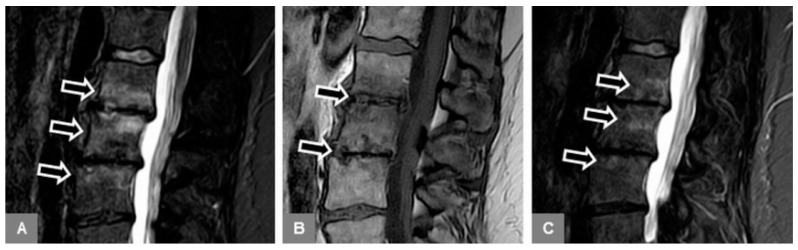
Modic type 1. A 52-year-old female patient with vertebral bone marrow edema on a sagittal STIR image at L1 to L3 (**A**, arrows), and with marked irregular, yet intact endplate contours and disc space narrowing on a sagittal T1-w image (**B**, arrows). There was a decrease of edema on a follow-up examination eight weeks (**C**, arrows) after the initial presentation.

The following inclusion criteria were used to select patients:

1. Patients with MT1 endplate changes (Figure 1):

Available, standard, unenhanced MR examination of the lumbar spine that yielded a tentative diagnosis of MT1, as well as follow-up MR imaging (two to eight weeks afterward) confirming the diagnosis, with mono- or multisegmental involvement, without osseous destruction and vertebral height decrease, and without severe paravertebral phlegmonous changes involving the paravertebral musculature.

Non-specific low back pain for at least three months, with or without signs of spinal segmental instability on clinical examination (pain pattern not attributable to a recognizable, specific pathology; maximum pain in the morning; morning stiffness for longer than 60 min; night-time pain; pain during back extension). 

**Figure 2:**
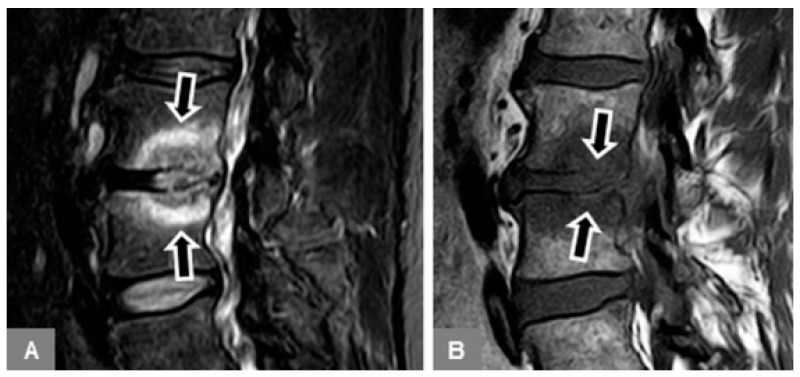
Early spondylodiscitis. A 63-year-old male patient with vertebral bone marrow edema on a sagittal STIR image at L3/L4 (**A**, arrows), with blurred, discontinuous endplate contours on a sagittal T1-w image (**B**, arrows).

2. Patients with early stage spondylodiscitis (Figure 2):

Available, standard MR examination, including contrast-enhanced images, of the lumbar spine that yielded a tentative diagnosis of early spondylodiscitis, defined as mono- or multisegmental involvement, without osseous destruction and vertebral height decrease, without severe paravertebral phlegmonous changes involving the paravertebral musculature, and without abscess formation. 

Clinical symptoms suggestive of spondylodiscitis: lumbar pain on heel strike, impaction, and percussion; pain on inclination and re-erection; constant lumbar pain worsening at night, with or without neurological deficits (leg weakness, paralysis, sensory deficits, radiculopathy, and sphincter loss). 

Increase of inflammatory parameters (C-reactive protein, leukocytosis, and/or erythrocyte sedimentation rate), with or without fever.

Positive proof of florid infection and/or pathogen, obtained by biopsy, needle aspiration, or blood culture.

**Figure 3:**
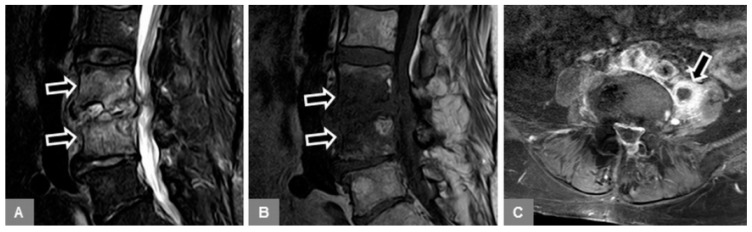
Advanced spondylodiscitis. A 79-year-old female patient with vertebral bone marrow edema on a sagittal STIR image at L3/L4 (**A**, arrows), with vertebral endplate destruction on a sagittal T1-w image (**B**, arrows), and left-sided, rim-enhancing psoas abscess formation on a transverse T1-w contrast-enhanced, fat-suppressed image (**C**, arrow).

3. Patients with advanced stage spondylodiscitis (Figure 3):

Available, standard MR examination, including contrast-enhanced images, of the lumbar spine that yielded a tentative diagnosis of spondylodiscitis, defined as mono- or multisegmental involvement, with or without destructive vertebral height decrease, with severe paravertebral phlegmonous changes involving the paravertebral fat tissue and musculature, and with abscess formation. 

Clinical symptoms suggestive of spondylodiscitis: lumbar pain on heel strike, impaction, and percussion; pain on inclination and re-erection; constant lumbar pain worsening at night with or without neurological deficits (leg weakness, paralysis, sensory deficits, radiculopathy, and sphincter loss). 

Increase of inflammatory parameters (C-reactive protein, leukocytosis, and/or erythrocyte sedimentation rate), with or without fever.

Positive proof of florid infection and/or pathogen, obtained by biopsy, needle aspiration, or blood culture.

Exclusion criteria included: vertebral fractures; spondylolysis; severe scoliosis and congenital vertebral abnormalities; tuberculous spondylodiscitis; sero-positive rheumatoid arthritis and sero-negative forms of spondyloarthritis; myositis; previous spinal trauma; previous spinal surgery or interventional therapy; pregnancy; and metastatic tumor diseases.

### 2.2. MR Imaging

The MR examinations of the lumbar spine were performed on a 3.0 Tesla MR unit (Philips Achieva; Philips Medical Systems, Best, the Netherlands) using a 15-channel spine coil. All MR scans were performed in the supine position and head-first on the scanner table, with the patient’s arms positioned beside the body. All patients had a standard wedge-shaped foam lower leg support (15 cm maximum height), which resulted in slight bending of the hip and knee joints. Sagittal MR images were oriented along the posterior edge of the vertebral bodies, covering the lumbar spine in a craniocaudal direction from the upper endplate of vertebral body Th11 to at least vertebral body S2. In the dorsoventral direction, the lumbar spine was visualized, including at least 5 cm of antevertebral soft tissue and the skin of the dorsum. In the lateral direction, the lumbar spine was visualized, including the transverse process on both sides. 

The 3.0 Tesla MR protocol to image the lumbar spine included the following MR sequences relevant to this study:

(a) A sagittal T1-weighted (w) fast-spin-echo (FSE) sequence (repetition time (TR), 500–650 msec; echo time (TE), 8 msec; flip angle, 90°; field of view (FOV), 300 × 300 mm; matrix, 800 × 800; in-plane resolution, 0.38 mm × 0.38 mm; slice thickness, 3 mm; interslice gap, 0.3 mm); (b) A sagittal T2-w FSE sequence (TR, 4000–4500 msec; TE, 105 msec; flip angle, 90°; FOV, 300 × 300 mm; matrix, 800 × 800; in-plane resolution, 0.38 mm × 0.38 mm; slice thickness, 3 mm; interslice gap, 0.3 mm); (c) A sagittal short tau inversion recovery (STIR) sequence (TR, 4250–5500 msec; TE, 70 msec; inversion time (TI), 180 msec; flip angle, 90°; FOV, 300 × 300 mm; matrix 528 × 528; in-plane resolution, 0.57 mm × 0.57 mm; slice thickness, 3 mm; interslice gap, 0.3 mm).

In addition, in all patients with spondylodiscitis, a contrast-enhanced sagittal T1-w FSE sequence and a transverse T1-w FSE sequence with selective fat supression were obtained. Please note that our retrospective evaluation was based on unenhanced MR images alone, because contrast-enhanced MR images were not available in all MT1 patients.

### 2.3. Evaluation

All the MR examinations were anonymized and randomly presented on a picture archiving and communication system (IMPAX, AGFA HealthCare, Bonn, Germany) to an MR imaging specialist (SFN, 16 years of experience in MR imaging), who was not aware of any patient data. For the assessment of the interreader agreement, a radiological fellow (USN, six years of experience in MR imaging) evaluated the same images in an independent reading session, and was also unaware of any patient data. For the assessment of the intrareader agreement, the images were re-evaluated by the MR specialist five weeks after the first evaluation. 

**Figure 4:**
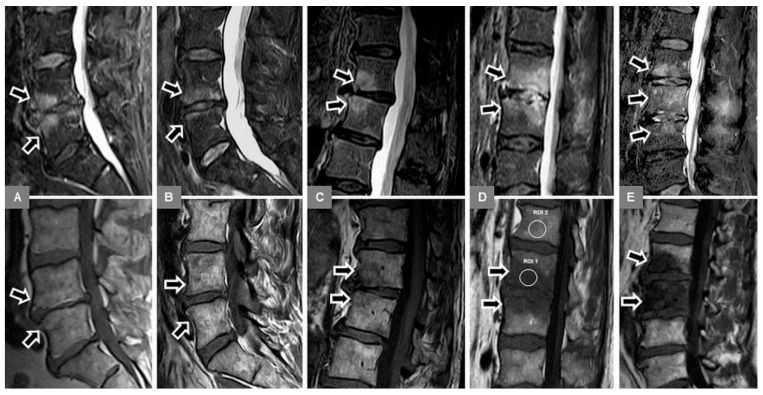
Visual assessment of the T1-w signal (bottom row of sagittal T1-w images, arrows), corresponding to the bone marrow edema (top row of sagittal STIR images, arrows), compared to normal bone marrow, in different patients with MT1 or spondylodiscitis. (**A**) A 53-year-old female patient with Modic type 1 and a hyperintense T1-signal at L4/5. This signal hyperintensity is explained by the overlap of bone marrow edema and fatty marrow conversion, thus additional Modic type 2 changes. (**B**) A 53-year-old female patient with Modic type 1 and isointense T1-signal at L4/5. (**C**) A 78-year-old female patient with Modic type 1 and mixed hyper-/hypointense T1-signal at Th12/L1. (**D**) A 63-year-old male patient with early spondylodiscitis and hypointense T1-signal (equal to the musculature) at L2/3. The region of interest (ROI) (1) at L2 exemplifies the measurement of the T1-signal intensity within the bone marrow edema area, and the ROI (2) within the adjacent unaffected vertebral body at L1. (**E**) A 66-year-old male patient with early spondylodiscitis and marked hypointense T1-signal (more hypointense than the musculature) at L2/3.

First, the whole stack of the sagittal STIR images was used to confirm the presence of bone marrow edema of the vertebral body, defined as an area of high signal intensity within at least the subchondral vertebral bone marrow adjacent to the endplate (Figure 4). The edema extent was subjectively assessed as a percentage of volume of the vertebral body (in increments of 10 from 10% to 100%), and edema was classified as mono- or multisegmental involvement. Any further evaluation, as detailed below, was then confined to the affected vertebral bodies and segments.

Second, a sagittal STIR image, showing the maximum hyperintense bone marrow edema changes, was manually selected, and the corresponding T1-w image was indicated by a reference tool. On this sagittal T1-w image, using a workstation-integrated software tool, a preformed circular region of interest (ROI) was manually adjusted to include an area of 200 pixels² and placed within the center of the visible signal changes, corresponding to the STIR-hyperintense edema area (Figure 4D). Furthermore, in an analogous manner, another ROI was manually adjusted and placed within the unaffected bone marrow of an adjacent lumbar vertebral body, which did not exhibit any signal abnormalities (Figure 4D). Thus, these T1-w signal intensity measurements were assessed to obtain the T1-w signal ratio of edema to unaffected vertebral bone. In each patient, single measurements were performed by both readers. Then, the T1-w signal changes, corresponding to the edema area, were also visually assessed, compared to unaffected bone marrow as follows: (a) hyperintense; (b) isointense; (c) mixed hyper-/hypointense; (d) hypointense (equal to paravertebral musculature); and (e) marked hypointense (more hypointense than paravertebral musculature) (Figure 4).

**Figure 5:**
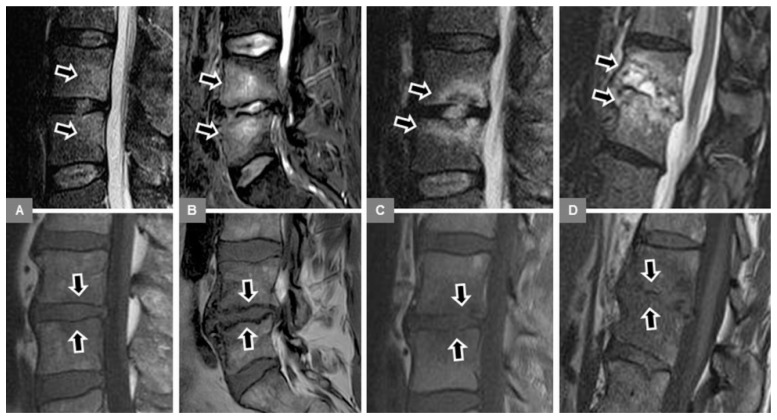
Evaluation of the vertebral endplate contour (bottom row of unenhanced sagittal T1-w images, arrows) in different patients with vertebral bone marrow edema (top row of sagittal STIR images, arrows), resulting from MT1 or spondylodiscitis. (**A**) A 54-year-old male patient with early spondylodiscitis and normal endplates at L3/4. (**B**) A 53-year-old male patient with Modic type 1 and irregular, but intact endplates and marked T1-w hypointense bony sclerosis, representing additional Modic type 3 changes at L4/5. (**C**) A 55-year-old male patient with early spondylodiscitis and blurred, discontinuous endplates at L3/4. (**D**) A 63-year-old male patient with advanced spondylodiscitis and endplate destruction with height decrease at Th12/L1.

Third, on unenhanced sagittal T1-w images, the appearance and integrity of the vertebral endplate contour (upper and lower), representing a thin hypointense, linear layer, were evaluated as follows: (0) normal intact appearance: continuous sharp linear contour; (1) irregular, yet intact appearance: irregular but sharp continuous contour; (2) blurred appearance: fuzzy discontinuous contour, without linear appearance; and (3) destructive appearance: irregular defect(s) with discontinuous contour, with or without vertebral body height decrease (Figure 5).

**Figure 6:**
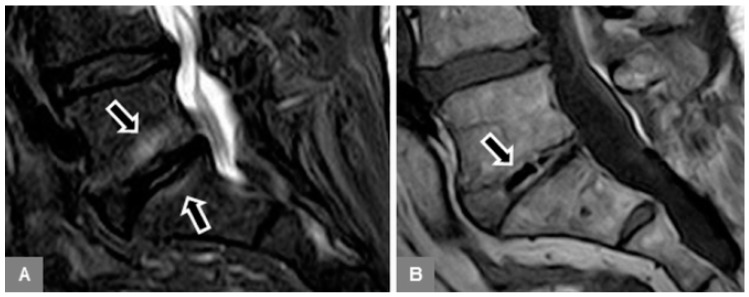
Intervertebral disc vacuum phenomenon in Modic type 1. A 73-year-old female patient with vertebral bone marrow edema on a sagittal STIR image at L5/S1 (**A**, arrows), with the vacuum phenomenon seen as a low signal intensity within the degenerated, height-reduced disc on a sagittal T1-w image (**B**, arrow).

Fourth, on unenhanced sagittal T1-w and T2-w images, the intervertebral disc was evaluated for the presence of a vacuum phenomenon (gas content), seen as low signal within the disc (Figure 6); and on sagittal STIR images, for the presence of a fluid-equivalent signal hyperintensity.

Fifth, unenhanced sagittal T1-w and T2-w images were used to identify possible additional Modic type 2 changes of the vertebral endplates and subchondral bone, seen as T1-w and T2-w signal hyperintensity (Figure 4A), which represented normal, red hemopoietic bone marrow conversion into yellow fatty marrow. These images were also used to identify possible additional Modic type 3 changes (T1-w and T2-w signal hypointensity), which represented subchondral bony sclerosis (Figure 5B).

### 2.4. Statistical Analysis

Statistical analysis was performed using software (IBM SPSS Statistics 25.0 for Windows; SPSS, Armonk, NY). A *p*-value equal to or less than 0.05 was considered to indicate a significant difference.

Descriptive statistics, including mean, median, minimum, maximum, standard deviation, and range, were calculated for the percentage extent of vertebral body edema and T1-w signal ratios of edema to unaffected vertebral bone, as observed in MT1, and early and advanced spondylodiscitis, including all measured values of affected vertebral bodies. Cross-tabulation was used to demonstrate the absolute frequencies and percentages of visually assessed T1-w signal changes, as well as of the upper and lower endplate contour assessment, as observed in MT1, and early and advanced spondylodiscitis. Taking into account that patients had multisegmental involvement, and thus, multiple measured values, for edema extent and T1-w signal ratios, differences were tested for statistical significance by using a mixed model analysis of variance; and for the visual T1-w signal changes and endplate contour by using a Chi-square test. 

Please note that, in this study, the assessment of the diagnostic accuracy (sensitivity, specificity, and accuracy) referred to the correct identification of MT1. A receiver-operating characteristic (ROC) analysis was performed, and the area under the time-intensity curve (AUC) was calculated to assess the optimal threshold, and thus, the diagnostic accuracy of the percentage of edema extent and the T1-w signal ratio of edema to unaffected bone to differentiate between MT1 and spondylodiscitis. In addition, 95% confidence intervals were calculated. Based upon cross-tabulation, the diagnostic accuracy, and the positive and negative predictive values of the contour evaluation, using the feature “irregular, yet intact” for the differentiation of MT1 and spondylodiscitis were assessed, considering the abnormal or most affected endplate in each individual. A logistic regression analysis was performed to assess the diagnostic accuracy of MR imaging in the differentiation of MT1 and spondylodiscitis, when combining the percentage of edema extent, T1-w signal ratio of edema to unaffected bone, and endplate contour. Furthermore, a multinominal logistic regression analysis was performed to demonstrate the percentages of correct classification of MT1, and early and advanced spondylodiscitis based upon the combined features of edema extent, T1-w signal ratio, and endplate contour. This regression analysis addressed MT1, and early and advanced spondylodiscitis separately without considering their differential diagnostic relationship.

Cross-tabulation was also used to demonstrate the absolute frequencies and percentages of vertebral body involvement at Th12 to L1, the vacuum phenomenon, the discal STIR hyperintensity, and any additional Modic type 2 and 3 changes in MT1, and early and advanced spondylodiscitis. Differences were tested for statistical significance using a generalized estimating equation (GEE).

Using the intraclass correlation coefficient (ICC), the intrareader and interreader agreement were assessed for the quantitative evaluation of the percentage of edema extent and the T1-w signal intensity measurements (continuous variables). Using weighted κ statistics, the intrareader and interreader agreement were assessed for the visual, qualitative evaluation of the vertebral endplate contour and the T1-w signal intensity within the edema area (categorical variables). 

## 3. Results

Based upon the aforementioned criteria, we included a total number of 91 patients. There were 39 patients with MT1 changes (20 women and 19 men; age range, 24–90 years; mean age, 59.6 years), and 52 patients with infectious spondylodiscitis (11 women and 41 men; age range, 31–81 years; mean age, 64.5 years), including 19 patients with early spondylodiscitis, and 33 with advanced spondylodiscitis. 

Of 39 patients with MT1, 28 had monosegmental and 11 multisegmental involvement. Of 52 patients with spondylodiscitis, 46 had monosegmental and six multisegmental involvement (three with early and advanced spondylodiscitis, respectively). Of 33 patients with advanced spondylodiscitis, seven had paravetrebral, 21 paravetrebral and epidural, and five epidural abscess formations.

In 18/52 patients with spondylodiscitis, there was florid infection shown by biopsy, needle aspiration, or blood culture, but without proof of a specific pathogen. In 34/52 patients, a specific pathogen could be identified: *Staphylococcus aureus* in 16; *Staphylococcus epidermidis* in two; *Streptococcus* species in four; *Escherichia coli* in four; *Pseudomonas aeruginosa* in four; *Enterococcus* species in two; and *Klebsiella* species in two patients.

### 3.1. Bone Marrow Edema Extent, T1-w Signal Ratios of Edema to Unaffected Bone, and Visual T1-w Signal Assessment

The descriptive statistics for the percentage of bone marrow edema extent and T1-w signal ratios of edema to unaffected bone are detailed in Table 1, based upon all measured values. The percentage extent of vertebral bone marrow edema showed statistically significant differences (*p* < 0.001) among MT1 (mean extent, 31.96%; standard error, 3.02), early spondylodiscitis (mean extent, 56.42%; standard error, 4.43), and advanced spondylodiscitis (mean extent, 91.84%; standard error, 3.38). The T1-w signal ratios of edema to unaffected bone of MT1 (mean ratio, 0.831; standard error, 0.019) were significantly different (*p* < 0.001) from early spondylodiscitis (mean ratio, 0.598; standard error, 0.027), and advanced spondylodiscitis (mean ratio, 0.613; standard error, 0.021); there was no significant difference (*p* = 1.000) between early and advanced spondylodiscitis. The visually assessed T1-w signal changes within the edema area of MT1 were significantly different (*p* < 0.001) from early spondylodiscitis, and advanced spondylodiscitis; there was no significant difference (*p* = 0.837) between early and advanced spondylodiscitis. A mixed hyper-/hypointense signal was seen in 72.4% of MT1, a hypointense signal in 47.5% of early spondylodiscitis, and a hypointense signal in 39.7% of advanced spondylodiscitis (Table 2).

### 3.2. Vertebral Endplate Contour

The endplate contours of MT1 were significantly different (*p* < 0.001) from early spondylodiscitis, and advanced spondylodiscitis; and between early and advanced spondylodiscitis as well (*p* = 0.015). The upper endplate was irregular, yet intact in 88.1% of MT1, blurred in 68.2% of early spondylodiscitis, and blurred in 68.6% of advanced spondylodiscitis; the lower endplate was irregular, yet intact in 94.9% of MT1, blurred in 68.2% of early spondylodiscitis, and blurred in 65.7% of advanced spondylodiscitis (Table 3). A normal endplate was found in up to 10.2% of MT1, in 18.2% of early and in 5.7% of advanced spondylodiscitis, respectively, and endplate destruction was seen in advanced spondylodiscitis only (Table 3).

### 3.3. Diagnostic Accuracy in the Differentiation Between MT1 and Spondylodiscitis

The ROC analysis revealed a percentage edema extent of ≤ 55% (sensitivity, 79.49% (31/39); specificity, 86.50% (45/52); accuracy, 84.62% (77/91)), and a T1-w signal ratio of edema to unaffected bone of ≥0.80 (sensitivity, 82.10% (32/39); specificity, 86.50% (45/52); accuracy, 84.62% (77/91)) as the optimal thresholds for identifying MT1, and thus, differentiating spondylodiscitis (Table 4). The presence of an irregular, yet intact endplate contour showed a sensitivity of 94.87% (37/39), a specificity of 94.23% (49/52), and an accuracy of 94.51% (86/91) for identifying MT1, and thus, differentiating spondylodiscitis, with a positive predictive value of 92.50% (37/40) and a negative predictive value of 94.23% (49/52). The combination of the percentage of edema extent, T1-w signal ratio, and endplate contour showed a sensitivity of 100% (39/39), a specificity of 94.23% (49/52), and an accuracy of 96.70% (88/91). The overall percentage of correct classification was 87.91% (80/91), including MT1, and early and advanced spondylodiscitis, based upon the combination of edema extent, T1-w signal ratio, and endplate contour (Table 5).

### 3.4. Site of Vertebral Body Involvement

Considering the vertebral bodies Th12 to S1, L3 (18.9%), L4 (23.3%), and L5 (24.8%) were most commonly involved, without statistically significant differences (*p* = 0.999) between MT1 and early and advanced spondylodiscitis (Table 6).

### 3.5. Appearance of the Intervertebral Disc

There were statistically significant differences for the discal vacuum phenomenon (*p* = 0.001) and abnormal STIR signal hyperintensity (*p* < 0.000) between MT1 and early and advanced spondylodiscitis (Table 7, section A). A vaccum phenomenon of the disc was most commonly seen in MT1 (25.4%), and an abnormal discal hyperintensity most commonly in advanced spondylodiscitis (88.6%).

### 3.6. Modic Type 2 and 3 Endplate Changes

Additional Modic type 2 (33.9%) and type 3 (30.5%) changes were most commonly found in MT1 patients. There were statistically significant differences for additional Modic type 2 (*p* < 0.001) and type 3 (*p* < 0.001) changes between Modic type 1 and early and advanced spondylodiscitis (Table 7, section B).

### 3.7. Reader Agreement

There was high reader agreement for the quantitative assessment of the percentage of edema extent (intra ICC = 0.843; inter ICC = 0.765) and the T1-w signal intensity measurements (intra ICC = 0.820; inter ICC = 0.760). There was also high reader agreement for the visual, qualitative assessment of the T1-w signal intensity within the edema area (intra weighted κ = 0.887; inter weighted κ = 0.770) and the vertebral endplate contour (intra weighted κ = 0.888; inter weighted κ = 0.825).

## 4. Discussion

At 3.0 Tesla state-of-the-art spinal MR imaging, this study systematically investigated imaging features for the differentiation between MT1 and early and advanced spondylodiscitis, all presenting with bone marrow edema. The endplate assessment on unenhanced T1-w images provided high diagnostic accuracy for identifying MT1, and thus, differentiating spondylodiscitis. This may be particulary useful in early-stage infection in the absence of distinctive features, such as vertebral destruction and abscess formation.

In our study, the vertebral bone marrow edema extent was evaluated by using fat-suppressed, fluid-sensitive MR images, which have been shown to reveal more MT1 changes, compared to the standard T2-w images used for their original description [1,35]. Detailed analysis was provided in addition to previous results that demonstrated MT1-edema mostly confined to the subchondral bone, and infectious edema predominantly extending to the opposite unaffected endplate [30]. In our study, the edema extent showed significant differences among MT1 (mean, ~32%), early (~56%), and advanced spondylodiscitis (~92%), thus, illustrating what has been learned from anecdotal evidence: the more edema, the higher the likelihood of infection. Statistically, when using a threshold of ≤55% edema extent, there was a sensitivity of 79.5% in identifying MT1, and hence, a limited ability to differentiate infection. These results also complement study findings of edema margin patterns [31,36]. Typically, subchondral edema in MT1 may show a sharply-marginated, band-like configuration, and diffuse infectious edema may show indistinct margins [1,3,31,32,36]. The latter, however, were found in 64% of patients with infection, and in 32% with degeneration, and may, therefore, not be reliable for differentiation [31].

This study included previous insights derived from MR imaging of pedal osteomyelitis that considered T1-w signal assessment of the edema area [37]. Fat-suppressed and T2-w sequences, including STIR images, may be overly sensitive in diagnosing edema, which is also seen in avascular necrosis, tumors, fractures, stress reaction, or neuropathic arthropathy [37]. Consequently, the interpretation of edema should include T1-w images, and thus, T1-w hypointensity in osteomyelitis [37]. Therefore, we quantified T1-w signal intensities within the edema compared to unaffected bone and visually qualified T1-w signal changes, which showed significant differences between MT1 and spondylodiscitis. This quantification, and thus, the signal ratios related to equipment-specific MR parameters may consequently impair its practical application. Both the signal ratios and visual assessment were not significantly different between early and advanced spondylodiscitis, alluding to their infectious pathogenesis. Visually, there were hypointense T1-w signals in 80.0% of early spondylodiscitis, but mixed signals in 72.4% of MT1. The latter may be explained by the reactive nature of edema, and, in addition, by overlapping Modic type 2 changes in 33.9% of MT1, which represented fatty marrow conversion that likely masked the expected T1-w signal decrease in edema. The additional Modic type 2 and 3 changes, most commonly seen in MT1 patients, also demonstrated the dynamic nature and possible mixed pattern of Modic changes [38].

Technically, unenhanced T1-w MR images, as currently and previously used, may be best suited for evaluating the vertebral endplate contours [27,28,29,30,36]. The T1-w phenomenon of endplate “pseudosparing,” which refers to an increased visibility of the endplate in infection due to chemical shift artifacts, can be avoided by selection of the craniocaudal phase-encoding plane [27,39]. The latter is customarily used at our institution. In contrast, T2-w images may be less useful for the evaluation of the endplate, which may be overstated because of marked chemical shift artifacts, depending on various sequence-inherent parameters. Reported in pedal osteomyelitis, diseased cortical bone “disappears” on unenhanced T1-w images and “reappears” more distinctly on T2-w and T1-w-enhanced images, also known as a “ghost sign” [40].

Vertebral endplate destruction is typical for spondylodiscitis [21,22,23,24,25,27,28,29,30]. However, in this study, endplates in MT1, and early and advanced spondylodiscitis were found to be normal in up to 10.2%, 18.2%, and 5.7%, respectively. Likewise, in another study, 15.9% patients with spondylodiscitis had intact endplates. Consequently, a normal endplate may not rule out infection.

In this study, among edema and T1-w signal assessment, an irregular, yet intact endplate contour showed the highest diagnostic accuracy (sensitivity, 94.9%; specificity, 94.2%) in the identification of MT1, thus differentiating spondylodiscitis. In this specific research setting, the combination of endplate contour, edema extent, and signal ratio improved the diagnostic accuracy again, with a sensitivity of 100%. Practically, the endplate evaluation seems most simple and effective. These endplate irregularities in the context of osteochondrosis, characterized by tiny endplate defects, associated with microfractures and fragmentations, have been termed erosive osteochondrosis, and underscore its active degnerative state [7,8,30,32]. Since the word “erosive” is also used in conjunction with spondylodiscitis, it was not applied in our own study. In our study, in MT1, contour blurring was found in 1.7% of endplates, versus in early spondylodiscitis, in 68.2%, which related to a hazy, discontinuous contour. Kwon et al. found endplate destruction in 9/11 patients with early spondylodiscitis, and endplate erosion in 11/14 patients with MT1, without providing further accuracy analysis [36]. These findings may refer to the time course of infection and the time point of an initial imaging diagnosis. Among the few studies that compared endplate contours in MT1 and spondylodiscitis, Stäbler et al. stated a definable T1-w endplate sensitivity of 87.8% in identifying MT1; the same authors found no definable endplates in patients with spondylodiscitis, of whom 78.3% already had abscess formations [30].

Both MT1 and spondylodiscitis most commonly affected L3 to L5, possibly explained by the lower lumbar biomechanical alignment and vascular anatomy, respectively [41]. Therefore, the involvement site does not point toward any specific diagnosis. Furthermore, our study included only patients with mono- or multisegmental edema, but not with single endplate involvement. The latter may also occur in seronegative spondyloarthritis, but is almost never seen in MT1 [42]. MT1 may possibly be unilaterally monosegmental, e.g., at the concave side of scoliosis [43].

In this study, discal STIR-hyperintense signal changes occurred in both MT1 and spondylodiscitis, with significant differences. In infection, this typically involves the entire disc space, with a loss of the intranuclear cleft, known as the hot disc sign, with disc hyperintensity and enhancement reported in > 90% of cases [27,44]. In contrast, in MT1, the fluid signal is induced by discal disruption and associated with the vacuum phenomenon [45,46]. Based on our data, a hyperintense disc (MT1, 20.3%; early spondylodiscitis, 81.8%) may tend to result from infection, whereas a vacuum phenomenon (MT1, 25.4%; early spondylodiscitis, 4.5%) rather points toward degeneration. However, this may not enable a confident differentiation, especially considering that spondylodiscitis occurs in older adults (mean age, 64.5 years), with disc degeneration per se. In fact, in the literature, the frequencies of disc hyperintensity as observed in MT1 and spondylodiscitis differ quite a bit, potentially related to the condition´s severity [27,30,36]. In one study, it was found in 2.6% of MT1 and 82.6% of spondylodiscitis with significant differences, and in another study, in over two-thirds of both entities with non-significant differences [30,36]. Disc enhancement has been previously seen without significant differences in either spondylodiscitis or MT1 [8,30,36].

As a result of it being largely available and a rapid alternative when MR imaging is unavailable or contraindicated, multidetector CT has been recently investigated for the assessment of suspected spondylodiscitis [47,48]. CT was accurate in demonstrating endplate and cortical destruction, with a sensitivity of 79% and a specificity of 100% in detecting acute spondylodiscitis [47]. CT showed 90% of paravertebral abscesses, but only 6% of epidural abscesses [47]. Consequently, MR imaging may be important to rule out such complications [47]. Moreover, MR imaging may identify the very early stages of spondylodiscitis, with edema but without obvious bone destruction versus active degeneration [28,29,36]. On CT-guided biopsy, a high probability of positive pathogen detection was found in non-sclerotic endplate erosions [48]. These erosions may correspond to MR endplate blurring or subsequent destruction. In turn, our MR feature of an irregular, but intact endplate, mostly seen in MT1, may suggest that the diagnostic assessment be directed toward the avoidance of biopsy.

Our study has several limitations, mostly due to its retrospective nature. First, this study included a total of 91 patients, but a limited number with early spondylodiscitis (*n* = 19). Second, biopsy or needle aspiration results were not available in MT1 patients, which were diagnosed based only upon clinical features, negative blood culture results, and imaging findings, including follow-up. Thus, this study did not provide insights into the debated infectious pathogensis of MT1 [9,10,11,12,13]. Third, information about a potential pre-treatment with medication, before being referred to our tertiary referral center, was not available in every single patient, nor were the actual therapy and outcome. Fourth, since this was a retrospective analysis, the MR parameters, particularly the TR values relevant for the T1-w signal intensity ratios, slightly differed within the study population, albeit in a close range of 500 to 650 msec. Furthermore, retrospectively, because of the lack of MR B1 mapping and image raw data, it was not possible to perform an intensity normalization to correct intensity non-uniformity or inhomogeneities that might influence an intensity quantification. Thus, the T1-w signal ratios, assessed in this study, should be interpreted with caution, and may not be directly applied to any other MR field strength or technical settings. Finally, since they were not part of routine MR imaging, advanced techniques were not included, such as previously investigated diffusion-weighted imaging, demonstrating a particular appearance, termed the claw sign, to identify MT1 [49]. Our study, however, included unenhanced standard images only, because contrast-enhanced images were not available in all MT1 patients. This shortcoming may be seem as an advantage, considering the debate on the brain deposition of gadolinium-based contrast media after repeated injections [50]. Despite the lack of known clinical effects, this issue may involve patients with a vague diagnosis of MT1 or early infection, who undergo several follow-up scans. In any case, a blurred or destructive endplate contour, suggesting infection, should prompt contrast application to check for further soft tissue involvement.

In conclusion, in patients with vertebral bone marrow edema, the presence of an irregular, yet intact T1-w endplate contour, provides high diagnostic accuracy for identifying MT1, with a sensitivity of 94.9% and a specificity of 94.2%. In contrast, the absence of an irregular, yet intact endplate contour is highly suggestive of infection, and particularly of early stage spondylodiscitis in patients without abscess formation. This simple and robust assessment of the endplate contour, on unenhanced standard MR images, may be applied by general and musculoskeletal radiologists, as well as by orthopedic specialists on a routine clinical basis to aid in the patient´s diagnostic and therapeutic management.

## Figures and Tables

**Table 1:** Descriptive statistics for the percentage of the extent of vertebral body bone marrow edema and the T1-w signal ratios of edema to unaffected vertebral bone.

		Percentage Edema Extent (%)	T1-w Signal Ratios
**Modic type 1**	**Mean**	31.83	0.82
	**Median**	30.00	0.82
	**Standard deviation**	23.66	0.14
	**Minimum**	5.00	0.53
	**Maximum**	100.00	1.22
	**Range**	95.00	0.70
**Early spondylodiscitis**	**Mean**	55.61	0.59
	**Median**	60.00	0.56
	**Standard deviation**	28.18	0.14
	**Minimum**	5.00	0.34
	**Maximum**	100.00	0.91
	**Range**	95.00	0.57
**Advanced spondylodiscitis**	**Mean**	91.60	0.61
	**Median**	100.00	0.61
	**Standard deviation**	18.26	0.13
	**Minimum**	10.00	0.29
	**Maximum**	100.00	0.96
	**Range**	90.00	0.66

**Table 2:** Visual assessment of the T1-w signal intensity within the bone marrow edema area, compared to normal, unaffected vertebral bone marrow.

		T1-w Signal Intensity					
		Hyperintense	Isointense	Mixed	Hypointense	Marked Hypointense	Total
**Modic type 1**	***n***	8	8	71	11	0	98
	**%**	8.2%	8.2%	72.4%	11.2%	0.0%	100.0%
**Early spondylodiscitis**	***n***	0	4	4	19	13	40
	**%**	0.0%	10.0%	10.0%	47.5%	32.5%	100.0%
**Advanced spondylodiscitis**	***n***	0	4	14	27	23	68
	**%**	0.0%	5.9%	20.6%	39.7%	33.8%	100.0%
	***n***	8	16	89	57	36	206
	**%**	3.9%	7.8%	43.2%	27.7%	17.5%	100.0%

**Table 3:** Contour changes of the upper and lower vertebral endplates.

		Endplate contour				
		Normal	Irregular	Blurred	Destructive	Total
		Upper endplate				
**Modic type 1**	***n***	6	52	1	0	59
	**%**	10.2%	88.1%	1.7%	0.0%	100.0%
**Early spondylodiscitis**	***n***	4	3	15	0	22
	**%**	18.2%	13.6%	68.2%	0.0%	100.0%
**Advanced spondylodiscitis**	***n***	1	1	24	9	35
	**%**	2.9%	2.9%	68.6%	25.7%	100.0%
	***n***	11	56	40	9	116
	**%**	9.5%	48.3%	34.5%	7.8%	100.0%
		**Lower endplate**				
**Modic type 1**	***n***	2	56	1	0	59
	**%**	3.4%	94.9%	1.7%	0.0%	100.0%
**Early spondylodiscitis**	***n***	4	3	15	0	22
	**%**	18.2%	13.6%	68.2%	0.0%	100.0%
**Advanced spondylodiscitis**	***n***	2	1	23	9	35
	**%**	5.7%	2.9%	65.7%	25.7%	100.0%
	***n***	8	60	39	9	116
	**%**	6.9%	51.7%	33.6%	7.8%	100.0%

**Table 4:** Receiver-operating characteristic (ROC) analysis of the percentage of edema extent and T1-w signal ratio of edema to unaffected bone for the differentiation between MT1 and spondylodiscitis.

	AUC	Standard Error	Significance *p*-Value	Confidence Interval	
				Lower Bound	Upper Bound
**Percentage of the extent of edema**	0.882	0.036	0.000	0.812	0.953
**T1-w signal ratio of edema to unaffected bone**	0.920	0.027	0.000	0.867	0.974

Area under the curve (AUC).

**Table 5:** Percentages of correct classification of MT1, and early and advanced spondylodiscitis based upon the combined features of edema extent, T1-w signal ratio, and endplate contour.

		Modic Type 1	Early Spondylodiscitis	Advanced Spondylodiscitis	Total
**Modic type 1**	***n***	39	0	0	39
	**%**	100.0%	0.0%	0.0%	100.0%
**Early spondylodiscitis**	***n***	2	12	5	19
	**%**	10.5%	63.2%	26.3%	100.0%
**Advanced spondylodiscitis**	***n***	1	3	29	33
	**%**	3.0%	9.1%	87.9%	100.0%
	***n***	42	15	34	91
	**%**	46.2%	16.5%	37.4%	100.0%

**Table 6:** Site of vertebral body involvement.

		Vertebral Body							
		Th12	L1	L2	L3	L4	L5	S1	Total
**Modic type 1**	***n***	1	6	11	17	23	28	12	98
	**%**	1.0%	6.1%	11.2%	17.3%	23.5%	28.6%	11.2%	100.0%
**Early spondylodiscitis**	***n***	1	3	6	8	10	8	4	40
	**%**	2.5%	7.5%	15.0%	20.0%	25.0%	20.0%	10.0%	100.0%
**Advanced spondylodiscitis**	***n***	2	6	10	14	15	15	6	68
	**%**	2.9%	8.8%	14.7%	20.6%	22.1%	22.1%	8.8%	100.0%
	***n***	4	15	27	39	48	51	22	206
	**%**	1.9%	7.3%	13.1%	18.9%	23.3%	24.8%	10.7%	100.0%

**Table 7:** Intervertebral disc vacuum phenomenon and discal STIR signal hyperintensity (section A). Additional Modic type 2 and 3 changes (Section B).

**A**		**Intervertebral Disc Appearance**						
		**Vacuum Phenomenon**				**STIR Signal Hyperintensity**		
		**Absent**	**Present**	**Total**		**Absent**	**Present**	**Total**
**Modic type 1**	***n***	44	15	59		47	12	59
	**%**	74.6%	25.4%	100%		79.7%	20.3%	100.0%
**Early spondylodiscitis**	***n***	21	1	22		4	18	22
	**%**	95.5%	4.5%	100%		18.2%	81.8%	100.0%
**Advanced spondylodiscitis**	***n***	35	0	35		4	31	35
	**%**	100.0%	0.0%	100%		11.4%	88.6%	100.0%
	***n***	100	16	116		55	61	116
	**%**	86.2%	13.8%	100%		47.4%	52.6%	100.0%
**B**		**Vertebral Endplate**						
		**Modic type 2**				**Modic type 3**		
		**Absent**	**Present**	**Total**		**Absent**	**Present**	**Total**
**Modic type 1**	***n***	39	20	59		41	18	59
	**%**	66.1%	33.9%	100%		69.5	30.5	100%
**Early spondylodiscitis**	***n***	22	0	22		22	0	22
	**%**	100.0%	0.0%	100%		100%	0.0%	100%
**Advanced spondylodiscitis**	***n***	34	1	35		35	0	35
	**%**	97.1%	2.9%	100%		100%	0.0%	100%
	***n***	95	21	116		98	18	116
	**%**	81.9%	18.1%	100%		84.5%	15.5%	100%
